# An interpretable machine learning framework for measuring urban perceptions from panoramic street view images

**DOI:** 10.1016/j.isci.2023.106132

**Published:** 2023-02-03

**Authors:** Yunzhe Liu, Meixu Chen, Meihui Wang, Jing Huang, Fisher Thomas, Kazem Rahimi, Mohammad Mamouei

**Affiliations:** 1 Informal Cities, Oxford Martin School, University of Oxford, Oxford OX1 3BD, UK; 2 Geographic Data Science Lab, Department of Geography and Planning, University of Liverpool, Liverpool L69 7ZT, UK; 3 SpaceTimeLab, Department of Civil, Environmental and Geomatic Engineering, University College London, London WC1E 6BT, UK; 4 Department of Occupational and Environmental Health Sciences, School of Public Health, Peking University, Beijing 100191, China; 5 MRC Centre for Environment and Health, School of Public Health, Imperial College London, London W2 1PG, UK

**Keywords:** Environmental sciences, Artificial Intelligence

## Abstract

The proliferation of street view images (SVIs) and the constant advancements in deep learning techniques have enabled urban analysts to extract and evaluate urban perceptions from large-scale urban streetscapes. However, many existing analytical frameworks have been found to lack interpretability due to their end-to-end structure and “black-box” nature, thereby limiting their value as a planning support tool. In this context, we propose a five-step machine learning framework for extracting neighborhood-level urban perceptions from panoramic SVIs, specifically emphasizing feature and result interpretability. By utilizing the MIT Place Pulse data, the developed framework can systematically extract six dimensions of urban perceptions from the given panoramas, including perceptions of wealth, boredom, depression, beauty, safety, and liveliness. The practical utility of this framework is demonstrated through its deployment in Inner London, where it was used to visualize urban perceptions at the Output Area (OA) level and to verify against real-world crime rate.

## Highlights

• An interpretable framework to extract urban perceptions from panoramic SVIs
• Using panoptic segmentation to identify human-recognizable visual elements
• Crowdsourced SVI pairwise comparisons are quantified via the Elo rating system
• Using feature importance and accumulated local effects to improve interpretability

## Graphical abstract

**Figure:**
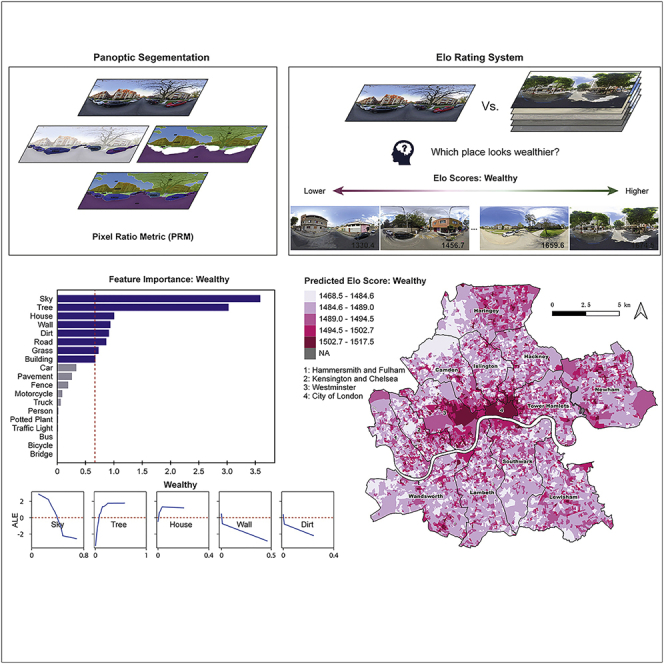

## Teaser

Geology; Artificial intelligence

## Introduction

As the environment where most human activities occur, cities can be characterized as an interchange hub for capital, logistics, labor, and information, shaping and influencing the lives of their residents from multiple perspectives.^1^^,^^2^ Numerous studies have shown that the physical appearance of cities plays a pivotal role in residents’ psychological feelings toward the urban built environment, consequently influencing their behaviors.^3^^,^^4^^,^^5^^,^^6^^,^^7^^,^^8^^,^^9^^,^^10^ Such human-perceived experience of the urban environment is also known as urban perception,^11^^,^^12^ together with urban identity, formulating important concepts in urbanism and urban design.^13^^,^^14^^,^^15^ Given the spatial heterogeneity and complexity of the urban built environment in terms of overall environmental quality and physical appearance, urban perceptions vary across different city areas. Therefore, research on urban perception offers a promising perspective that assists urban analysts in gaining insights into urban morphology and metabolism and the way residents perceive their living neighborhood areas, facilitating evidence-based policymaking in urban planning and regeneration.

Gathering information about visual surroundings from the urban built environment and evaluating their influences on human perceptions have a long research history.^7^^,^^16^^,^^17^^,^^18^^,^^19^^,^^20^ However, most previous studies relied on traditional data collection approaches, such as field surveys, questionnaires, and interviews, which are costly, error-prone, and time-consuming. As such, these studies encountered challenges in knowledge discovery and generalization, particularly for large-scale urban regions, due to the lack of the fine-granularity and high throughput of the investigation methods.^21^^,^^22^^,^^23^ With the proliferation of Street View Images (SVIs) data, such as Google Street View (GSV), Baidu Total View, Naver Street View, and Mapillary, and constant breakthroughs in machine learning and computational capacity, recently, SVIs have acquired significant traction in related research.^10^^,^^24^^,^^25^^,^^26^^,^^27^^,^^28^ Featuring its wide availability, adequate sample size, and consistent spatial granularity, SVI has yielded an unprecedented opportunity to characterize the visual and morphological properties of urban interior environments more accurately and comprehensively. Many studies have employed SVIs as the primary source of urban data and applied extracted visual elements to a plethora of urban problems, such as transportation and accessibility research^29^^,^^30^ and socioeconomic studies.^31^^,^^32^

Moreover, since SVIs enable human perspective research, namely, portraying detailed objects in the urban environment using a view angle comparable to the human eye, they have emerged as a promising data source for inferring urban perceptions. The overarching goal of this research domain is to develop a model that can measure and map the urban perceptual attributes of SVIs.^24^^,^^33^ As such, external surveys were frequently used in related studies to establish a connection between objective urban scenery and subjective human perceptions. For instance, to measure the visual quality of streets on a large scale, Ye et al.^34^ designed a rating platform to gather urban design experts’ preferences on the design elements based on Baidu SVIs in Shanghai. By inviting online volunteers to increase public participation, Salesses et al.^35^ extracted urban perceptions from massive pairwise comparisons of SVIs in the US and Austria to evaluate their impacts on socioeconomic outcomes. Following this work, Dubey et al.^36^ developed a GSV-based web interface using a massive online crowdsourcing strategy to extend the surveying scope to 56 cities globally, introducing the well-known MIT Place Pulse 2.0 dataset covering six perceptual dimensions—safe, lively, boring, wealthy, beautiful, and depressing. Facilitated by the data availability, globally, numerous studies have been undertaken to capture urban perceptions through utilizing various machine learning and computer vision techniques, including but not limited to support vector machines,^23^^,^^37^ artificial neural network,^34^ multiple linear regression,^21^ random forest,^22^ and deep learning.^33^^,^^38^

Although the above-mentioned studies have made great efforts in measuring urban perceptions from SVIs with the help of advancing deep-learning techniques, several research gaps still exist, limiting their practical utility as planning support tools for informing urban planning. First and foremost, most existing studies’ analytical frameworks lack interpretability owing to their end-to-end structure and “black-box” nature. The limited interpretability manifests in two aspects: feature interpretability and result in interpretability. First, visual features derived from a traditional deep-learning model are difficult for humans to grasp, limiting the interpretability of the results. These visual features are usually compiled by complex image patterns representing the most salient characteristics of the image, which are typically unrecognizable to humans and only discernible to machines.^39^ Although the deep-learning model may maintain the minutiae of the images to increase the prediction accuracy, its practical value is limited due to low feature interpretability. Second, since the image-to-perception structure (a.k.a., an end-to-end structure) is the commonly used structure of the urban perception model, the mechanism that transforms the input (i.e., SVI) into the output (i.e., indicators of urban perceptions) is still concealed in a “black-box” due to its complexity. For example, the visual features extracted from the SVI are usually embedded in the model pipeline and are not readily retrievable. Consequently, connections between visual features and urban perceptions are obfuscated in most related studies, diminishing their effectiveness in urban planning practice that requires valid evidence.^40^ Due to the restricted result interpretability, it may be difficult for decision-makers to answer concerns such as how to increase the perception of safety (or beauty) in a given neighborhood via urban regeneration and the provision of infrastructures.

The second noticeable research gap is that most urban perception research using SVIs lacks a relatively integrated perceptual representation of the urban environment due to the model input and the result presentation. In terms of the inputs, many studies utilized normal view SVI captured at the predetermined observation points to investigate urban perceptions. However, such SVI may be limited to certain angles or orientations, rendering it incapable of representing the whole viewshed of the given point. Although some studies have attempted to overcome this limitation by using multiple SVIs with different view angles at the same observing position,^23^ perceptions contained in the SVI were extracted separately, resulting in fragmental urban perceptions. As for the result presentation, related studies have overwhelmingly used road networks as the carrier to visualize the modeling outcomes.^21^^,^^22^^,^^23^ In these works, each segment of the road network displays a perceptual score calculated by spatially averaging the SVIs present at the segment. Although this geometric representation is straightforward and detailed since the observation points for capturing SVIs are set along the road, the complexity of the urban road network may prevent urban planners from readily deriving comprehensive information about urban perception in the related urban area. Additionally, given that most available urban data (e.g., census) are not provided at very fine geography due to geoprivacy protection,^41^^,^^42^ the street-level representation may limit the integration of urban perception research with other area-level urban data.

In this context, this study proposes an interpretable analytical framework for extracting neighborhood-level urban perceptions from panoramic SVIs (scoured from GSV), aiming to address the above-mentioned research gaps. Briefly, the core of this proposed framework is a random forest (RF) predictive model trained by using visual and perceptual features extracted from the MIT Place Pulse dataset, considerably improving interpretability in model training and result representation with prediction accuracy retained. This research provides two distinct contributions to the existing literature. First, instead of using multiple normal view SVIs at an observation point, we directly acquire panoramic SVIs (i.e., 360° photos) from the GSV platform, providing a holistic understanding of urban physical surroundings and urban perceptions from the perspective that is akin to a human. Moreover, in terms of the result representation, we substitute commonly used street-level visualization with neighborhood-level aggregation, emphasizing the importance of the urban neighborhood^28^ and also enhancing the research’s integrability for further studies in urban analytics. Second, instead of following the widely used end-to-end framework, we structure the workflow step-by-step to enhance the research interpretability. This workflow is mainly achieved by integrating the outcomes of both panoptic segmentation (i.e., independent variable) and the Elo rating approach (i.e., dependent variable) into the random forest regression. This architecture can considerably boost the model and the feature interpretability since the inputs are human recognisable, the outcomes of each intermediate step are retrievable, and the associations between inputs and outputs are investigable and interpretable. Furthermore, as the input variables (i.e., visual elements) are human-recognizable and their contributions to urban perceptions can be explored through the built-in feature importance function and accumulative local effect, the model transparency can be further amplified, hence improving its practical applicability.

The practical utility of this framework is exemplified in its implementation in the case study area (i.e., Inner London) with the association of real-world crime rate data for further verifying its applicability. To the best of our knowledge, this article is the first analysis using panoramic SVIs to explore neighborhood-level urban perceptions at a large scale in a UK city region, and it is also the first deployment of the urban perception model based on the MIT Place Pulse data in the UK context. The developed model is used to predict urban perception scores of the 122,733 panoramic SVIs captured within the Inner London area. Six dimensions of urban perceptions—wealthy, boring, depressing, beautiful, safe, and lively, are predicted and mapped at Output Area (OA) level—the smallest census geography in the UK. Additionally, the applicability of the predicted urban perception was verified by correlating it with real-world crime statistics, further demonstrating the outreaching potentiality of neighborhood-level urban perceptions.

## Results and discussion

### Random forest accuracy assessment

Table 1 presents six modeling results of the RF, composited by R^2^ and MAE for each of the perceptual dimensions. The overall average R^2^ is above 0.45, with the highest 0.52 in the prediction of safe perception, denoting that more than 45% of the variance in the observations can be explained by these 19 variables. The MAE for each perceptual dimension is less than 23, with the lowest MAE (12.00) reported for predicting boredom. Since the K-factor was set to 32 in the Elo rating system, suggesting that the maximum possible score for a single pairwise comparison is 32, the MAE of the trained RF model falls within a reasonable range.

**Table 1:** R squared and mean absolute error by perceptual dimension

	Beautiful	Safety	Wealth	Lively	Depressing	Boring
R^2^	0.49	0.52	0.48	0.51	0.51	0.45
MAE	16.30	21.22	14.35	16.70	14.57	12.00

### Random forest feature importance

**Figure 1:**
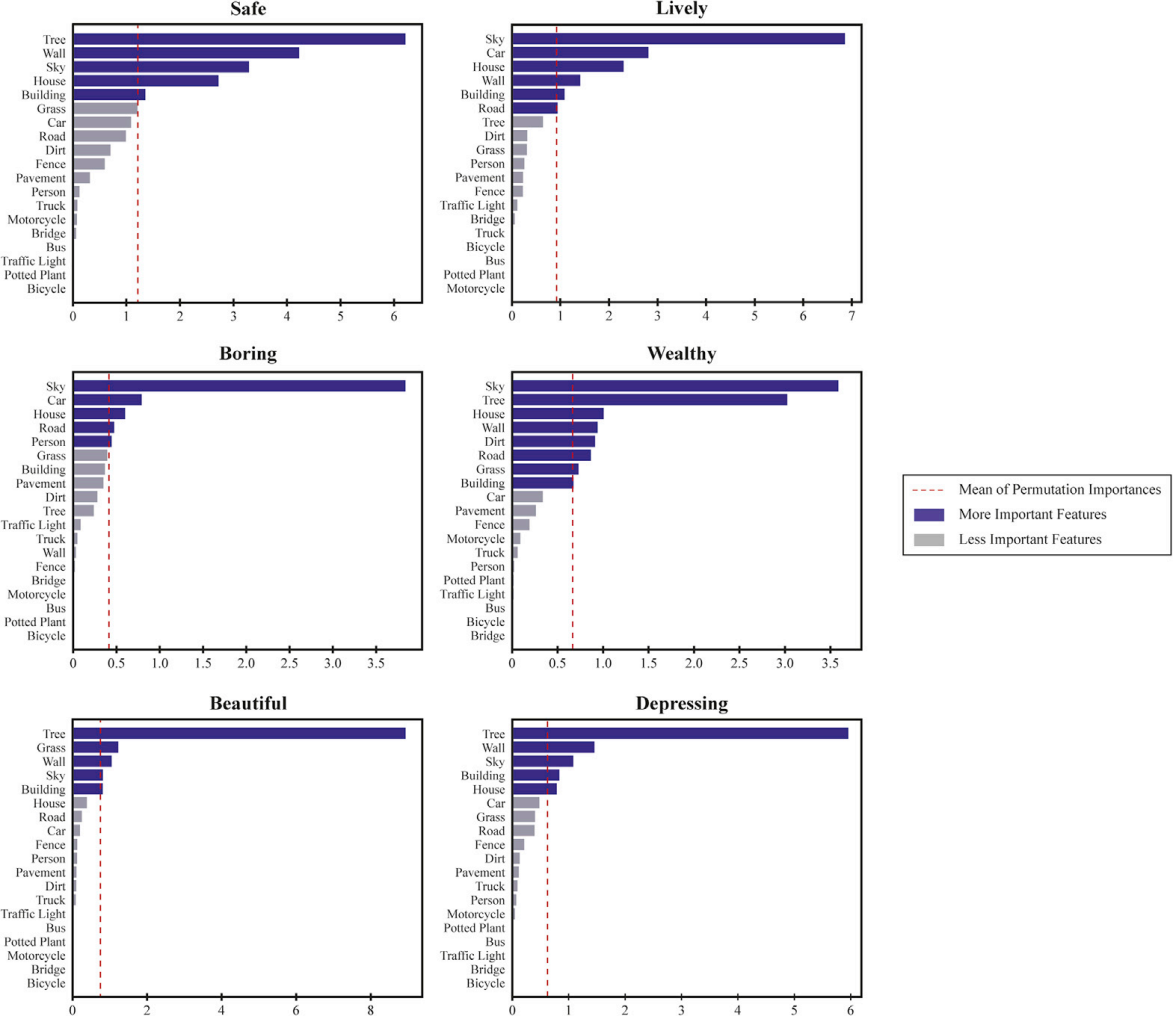
Feature importance for each dimension of urban perceptions

Figure 1 contains a series of bar charts illustrating the permutation feature importance (PFI) distribution of visual elements at each of the urban perception dimensions. The length of the bar depicts the value of the PFI, and the red dotted line is the average PFI, which is commonly used as a threshold for determining which variable is relatively more important for modeling. Overall, we noticed that the visual elements which are deemed more important to the RF prediction differ across six perceptual dimensions. For instance, “Car” and “House” are regarded as more significant predictors of the “Lively” dimension, whereas they are not as important as “Tree” and “Grass” in predicting the “Beautiful” dimension. Similarly, “Tree” and “Wall” are important contributors to predicting “Depressing” perception, whilst their importance is negligible in the “Boring” dimension. In addition to such heterogeneity, some visual elements remain prominent in the majority of urban perception predictions. For example, the area ratio of “Sky” is regarded as an important contributor in all six perceptual dimensions, whilst its contribution ranking varies across dimensions; Other than the “Lively” dimension, the area ratio of “Tree” significantly contributes to the model prediction in the rest of perceptual dimensions. In contrast, some visual elements, such as “Traffic light,” “Bus,” “Bicycle,” and “Bridge,” are negligible since their contribution is not evident in urban perception predictions. This may be attributable to their small quantity ratio in the SVI dataset, although the visual elements for predicting urban perceptions had been pre-filtered based on their occurrence frequency during the visual element extraction stage.

The overall results of Figure 1 are in accordance with earlier findings or theories in the existing literature. For instance, “Car,” “Building,” “Road,” and “House” are essential in the provision of a lively urban environment, which is in line with Jacob’s statement to promote the liveliness of a city.^43^ Objects associated with urban greenery, such as “Tree” and “Grass,” are significant in the majority of urban perception dimensions, especially the perception of beauty. This result is consistent with the perceptual functions and aesthetic benefits of urban vegetation discussed in^5^ and also aligns with Olmsted’s theory of integrating ecosystems into urban settings.^44^ Furthermore, with the exception of the “Boring” dimension, we observed that the other five dimensions of urban perceptions are sensitive to the areas of “Wall” in SVI, typically for the sensation of safety. This finding is consistent with the empirical results of^23^ and,^21^ where plethoric solid walls or similar large obstructions contribute to the reduction of public space visibility and permeability,^45^ resulting in reduced informal surveillance and “eyes on the street.”^46^

### Random forest interpretation

**Figure 2:**
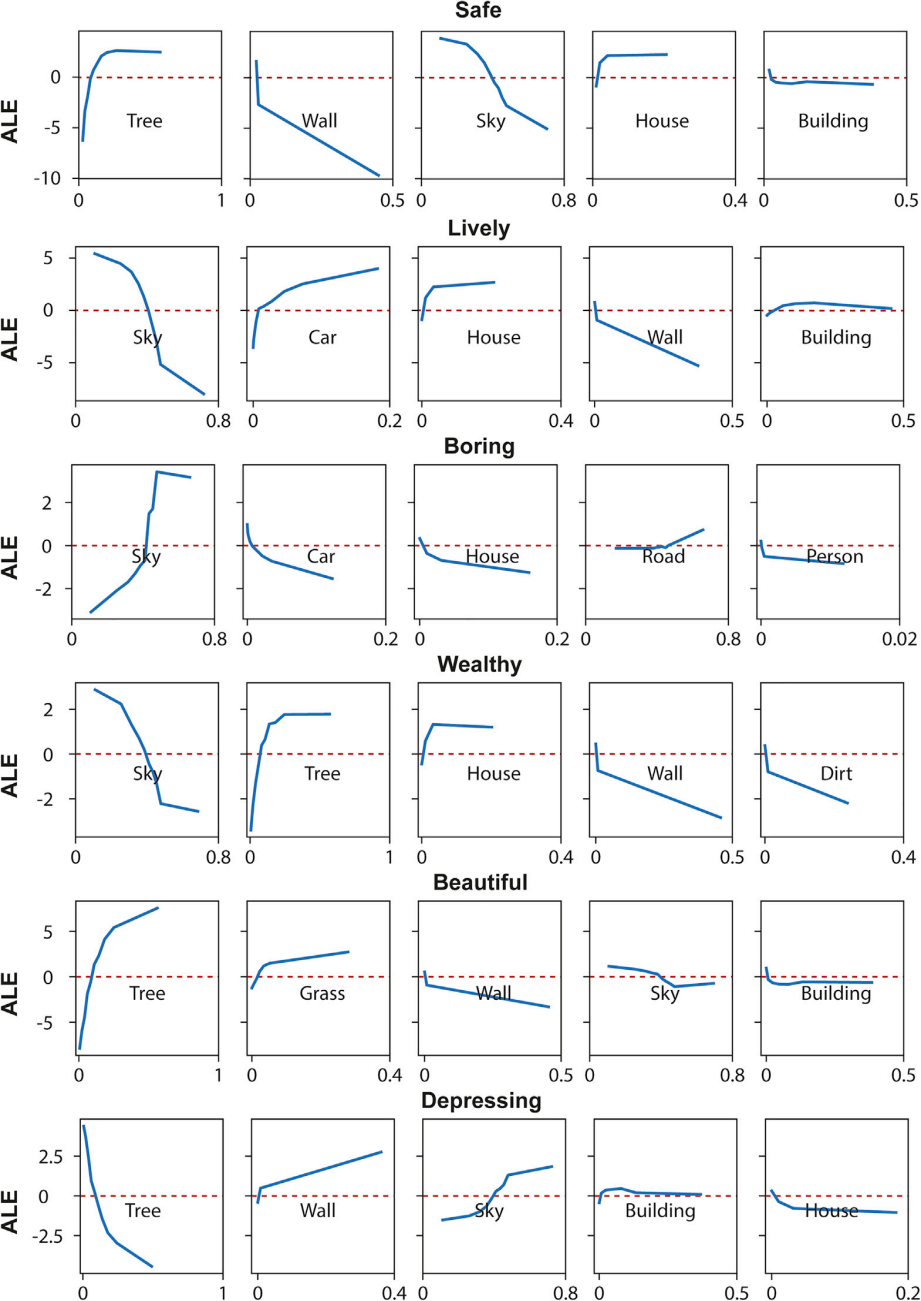
Accumulated Local Effects (ALE) plots for each dimension of urban perceptions

Figure 2 depicts a series of ALE plots illustrating how the top five important visual features influence each dimension of urban perception predictions. The x and y axes reflect the area ratio of each feature and zero-centered ALE values determined by Elo ratings of each urban perception, respectively. The overall findings presented in Figure 2 are consistent with common sense. For example, the area ratio of “Car” and “House” is positively associated with the “Lively” dimension; “Dirt” is negatively related to the prediction of the “Wealthy” dimension; “Tree” and “Grass” have a positive association with the “Beautiful” dimension. Given that the visual features in the ALE plots are arranged from left to right according to their importance, either “Tree” or “Sky” often plays the most crucial role in predicting all six dimensions of urban perception. When both features co-occur in the same dimension of urban perceptions, they have opposite effects on the prediction results. For instance, in the “Depressing” dimension, both “Tree” and “Sky” are recognized as important predictors; in general, the increasing area ratio of “Tree” will reduce the “Depressing” Elo score, while the increasing area ratio of “Sky” will enhance the sense of depression of the streetscape. Apart from the “Boring” dimension, “Wall” plays an important role in urban perception predictions, indicating that human perceptions are sensitive to visual obstacles set in the streetscape. With the additional information supplied by the ALE plots, “Wall” is negatively associated with all the remaining dimensions of urban perception and positively associated with the “Depressing” dimension. This suggests that the higher area ratio of “Wall” may diminish the streetscape”s impression of security, richness, vitality, and beauty while increasing the sensation of depression.

More detailed and specific interpretations can be given by examining the ALE plots by each dimension of urban perceptions. Taking the “Wealthy” dimension as an example, “Sky,” “Tree,” “House,” “Wall,” and “Dirt” are the top five important visual elements. “Sky” is negatively related to the perception of wealth, while its impact becomes much weaker until its share exceeds 50%. This pattern indicates that the sense of the place”s wealth is beneficial from the compact streetscape design since the sky openness might be decreased. “Tree” is positively linked to the wealthy perception, meaning that increasing the proportion of “Tree” in the streetscape will also increase the degree of people feeling wealthy about the place; however, after around 30% tipping point, increasing the share of “Tree” will become irrelevant thus does not increase the “Wealthy” attribute of the place. A similar pattern has been identified in the “House” visual element: the positive association is diminished after the 5% tipping point. As for “Wall” and “Dirt,” decreasing the ratio of these two visual elements will considerably increase the urban perception of wealth as they are both negatively associated with the “Wealthy” perception.

### Visualizing neighborhood-level urban perceptions

**Figure 3:**
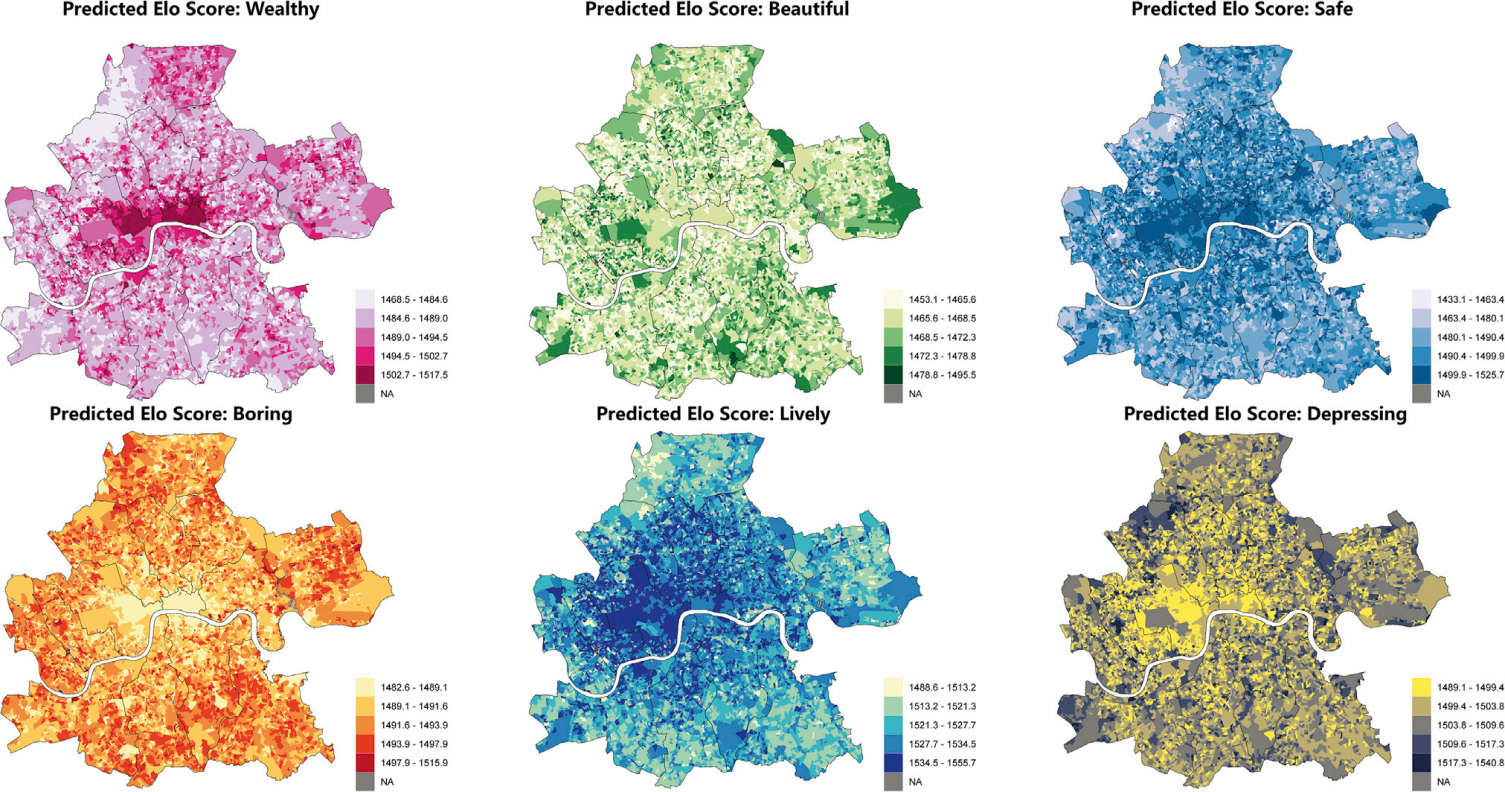
OA-level predicated Elo scores in Inner London by six perceptual dimensions

The obtained SVIs were converted into PRM through PS and then imported into the trained RF model. The predicted Elo scores were assigned to each SVI, followed by the neighborhood-level aggregation. Figure 3 contains a series of maps showing the spatial distribution of the predicted urban perceptions at the OA level for Inner London areas. OAs with “NA” value were attributed to the data availability, indicating that the given OA does not contain adequate SVIs (or no SVI) for spatial aggregation.

**Figure 4:**
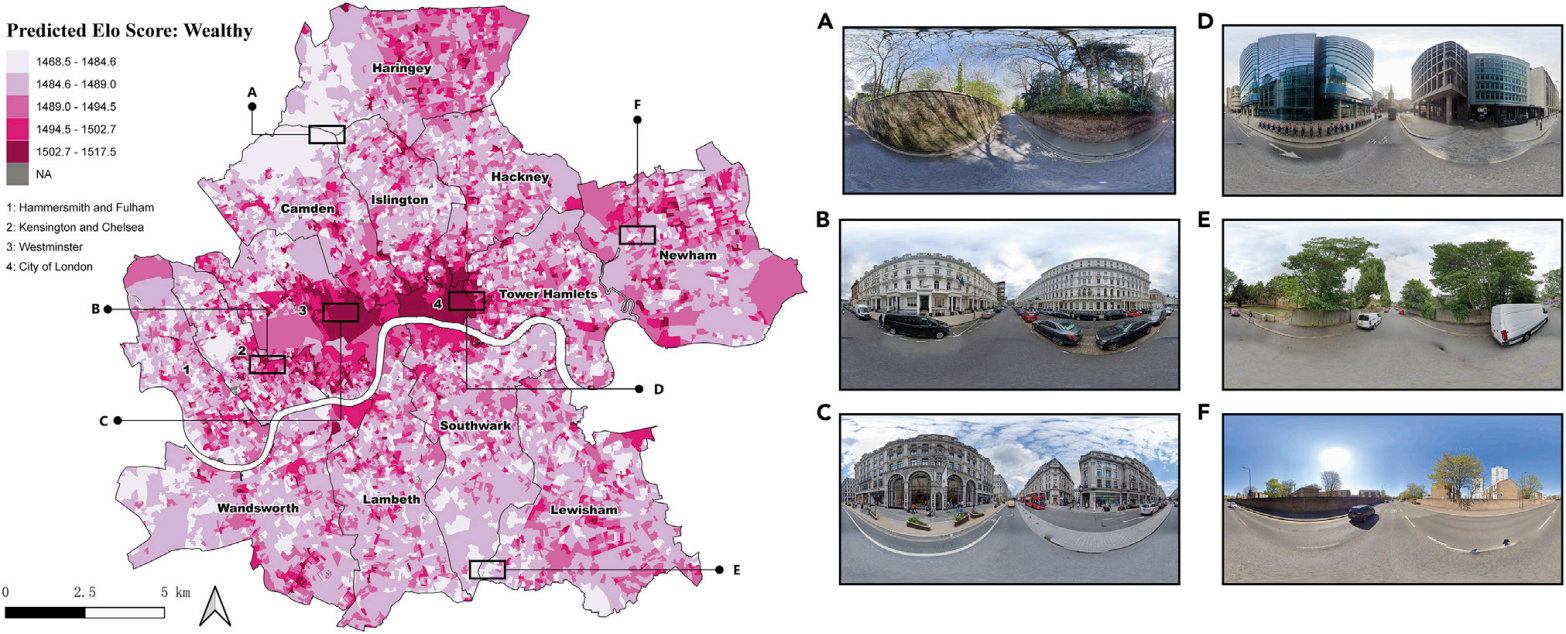
Spatial distribution of predicted “Wealthy” perception in Inner London at OA level

**Figure 5:**
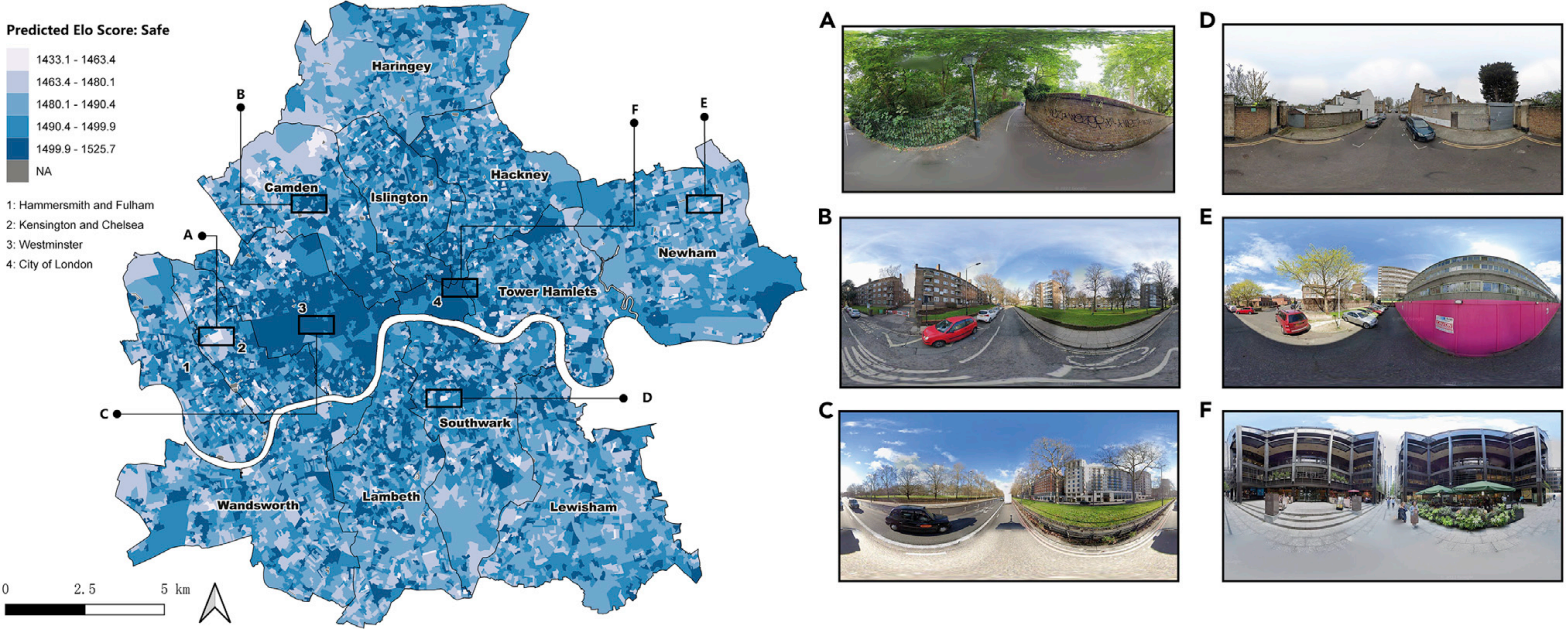
Spatial distribution of predicted “Safe” perception in Inner London at OA level

Figure 4 depicts a closer view of the spatial distribution of the predicted “Wealthy” perception in Inner London. The overall spatial distribution pattern is consistent with common sense. OAs with relatively higher Elo scores, namely, more affluent areas, are mainly clustered in Kensington and Chelsea (Area B), high streets in Westminster (Area C), and in the central business districts in City of London (Area D). Neighborhoods located at the outskirts of Inner London, especially northern Camden and western Haringey, are perceived as less wealthy from their streetscapes. A similar distribution pattern is also identified in the “Safe” dimension (Figure 5), in which most OAs located in Westminster (e.g., Area C) and City of London (e.g., Area F) provide an adequate sense of safety to their residences. From the representative SVIs captured in areas with relatively high Elo scores, visual permeability is one of the positive contributors to the perception of safety. This statement can also be manifested by the lower score OAs whose streetscapes are usually blocked by multiple obstacles, such as walls, fences, and vegetation (e.g., Areas A, D, and E).

### Applicability verification

The association between crime rate and the six dimensions of predicted urban perception is measured by Spearman's rank correlation coefficient (Table 2). Overall, with the exception of the “Beautiful” dimension, we identified weak correlations between crime rate and the other dimensions of urban perceptions. Such weak correlation might be because of the complexity of crime as they are typically impacted by multidimensional factors such as gender, age, education, and culture,^47^ although urban perception is one of the factors in committing crimes.

**Table 2:** Correlation verification between crime rate and six urban perceptions

	Wealthy	Safe	Lively	Depressing	Boring	Beautiful
Coefficient (r)	0.35	0.18	0.26	−0.14	−0.30	−0.04

Among these weak correlations, the “Wealthy” and “Boring” dimensions witness a relatively higher correlation coefficient, respectively exhibiting a positive (0.35) and a negative (−0.30) correlation with the theft crime rate, suggesting that theft crime is more likely to occur in areas where people feel wealthier and less bored. Such association can be further explained by the primary spatial distribution of these areas (Figure 3), namely, Westminster and City of London, containing a large agglomeration of tourist attractions, prominent landmarks, and commercial centers. The findings are in line with common sense: thieves are more likely to steal pedestrians’ belongings or commit shoplifting in densely populated areas with high population flow.

Surprisingly, the correlation coefficient between the crime rate and the “Safe” dimension is just around 0.18, namely, a negligible correlation, suggesting places perceived as safe does not necessarily correspond with a low actual crime rate and vice versa. A similar negligible correlation is also identified in the “Depressing” dimension. This might be because people are more likely to avoid visiting or remaining in areas that convey a sense of insecurity or depression. Such rejection reaction may reduce the likelihood of crowd gathering or heighten people’s awareness of self-protection, either or which is detrimental to criminal activity, especially street stealing. Conversely, perceptually safer or lesser depressing venues may draw more visitors, raising the potential of crowding, speeding up population flow, and decreasing people’s alertness, sometimes making individuals more vulnerable. This finding is in line with the “mismatching” between urban crime and the perception of safety discussed in.^48^

### Conclusion

The increased availability of SVI data and the ongoing development of deep learning techniques have enabled urban analysts to understand a large-scale urban environment from a human perspective. Numerous related studies have made significant efforts to extract human perceptions from visual elements of the urban physical environment. However, the analytical framework used in most existing studies lacked interpretability in model training, feature interpretation, and result explanation due to the “black-box” effect, limiting their utility as a planning support tool for evidence-based decision-making.

In this context, we proposed an interpretable analytical framework to automatically extract neighbourhood-level perceptions from urban streetscape shown in SVIs concerning six perceptual dimensions, namely, wealthy, boring, depressing, beautiful, safe, and lively. The proposed framework utilized the MIT Place Pulse data as a practical example and was structured by five main stages, including 1) Panoramic SVI acquisition and cleaning; 2) Visual element extraction; 3) Perception Elo rating; 4) Urban perceptions prediction; and 5) Neighbourhood-level aggregation.

By emphasizing feature and result interpretability, this framework contributed to bridging the research gaps in the existing literature. The feature interpretability was represented in the extraction of human recognizable visual elements generated by the FPN-based panoptic segmentation. The result interpretability was achieved predominantly by developing the RF regression model that utilises the visual elements as the intermediate to conduct urban perception predictions. The interpretability was further amplified by the analysis of feature importance and the ALE plots, which clarified the association between human-recognizable visual elements and each dimension of urban perceptions.

The practical value of this proposed framework was manifested in its implementation in the Inner London case study. To the best of the authors’ knowledge, this is the first attempt to analyze and map the spatial distribution of the neighborhood level (i.e., OA) urban perceptions in the UK context. The experimental findings demonstrated that the proposed framework is a cost-effective and accurate way of predicting human perceptions of large-scale metropolitan areas and may significantly enhance our knowledge of how humans perceive the urban physical environment. Additionally, the applicability of the predicted urban perception was verified by correlating it with real-world crime statistics, further demonstrating the outreaching potentiality of neighborhood level urban perceptions.

### Limitations of the study

The presented framework is extendable in several ways. In terms of the practical application, we suggest that using a bespoke local SVI dataset (i.e., panoramic images acquired in the case study area) to train the urban perception model is one direction of future research that might benefit the outcomes’ quality. Since the MIT Place Pulse data contain SVIs across the globe ^35^^,^^38^, urban perceptions extracted from the physical environment vary significantly. In the dataset, for example, gloomy slums in low-income countries mix alongside thriving high streets in developed countries. As such, the model trained with such data may not effectively discern the nuances of urban perceptions in a given city, especially if the urban streetscape lacks significant contrast. Moreover, because the nature of the urban perception prediction is based on a subjective crowdsourcing assessment that may be impacted by people’s socioeconomic and cultural backgrounds, a place with positive urban perceptions in one region of the world may have the opposite impression in other regions.^22^ A bespoke SVI dataset evaluated by local people with a similar sociodemographic composition in the same way as MIT Place Pulse might address such problems and help decision-making in local urban planning.

## STAR★Methods

### Key resources table

**Table:** 

REAGENT or RESOURCE	SOURCE	IDENTIFIER
**Deposited data**		

MIT Place Pulse 2.0 image database	MIT Media Lab	https://figshare.com/articles/dataset/Place_Pulse/11859993
OS MasterMap Highways Network - Paths	Ordnance Survey	https://digimap.edina.ac.uk/roam/download/os
OS MasterMap Highways Network - Roads	Ordnance Survey	https://digimap.edina.ac.uk/roam/download/os
2011 London Statistical Boundary data (Output Area, Boroughs)	Greater London Authority	https://data.london.gov.uk/dataset/statistical-gis-boundary-files-london
2015-2019 Crime data	Single Online Home National Digital Team	https://data.police.uk/data/

**Software and algorithms**		

Street View Download 360	Google Maps	https://svd360.istreetview.com/
Detectron2	Facebook	https://github.com/facebookresearch/detectron2/tree/main/configs/COCO-PanopticSegmentation
RStudio version 2022.02.4	posit	https://posit.co/download/rstudio-desktop/
Python version 3.9.7	Python Software Foundation	https://www.python.org/
QGIS version 3.22.5	Open-source software	https://qgis.org/en/site/

### Resource availability

#### Lead contact

Further information and requests should be directed to and will be fulfilled by the lead contact Yunzhe Liu (yunzhe.liu@wrh.ox.ac.uk).

#### Material availability

This study did not generate new unique materials.

### Method details

#### MIT Place Pulse dataset

Place Pulse (1.0 and 2.0), initiated by MIT Media Lab in 2013,^35^ is a typical crowdsourcing online data collection platform utilised to collect human perception of urban street appearance. On this platform, invited online volunteers are firstly presented with two randomly sampled street view images (powered by Google Street View) side-by-side, and then they are asked to justify the ‘winning images’ from the pairwise comparison according to the given questions. The body of the question is compiled by ‘which place looks more x?’ where x can be one of the six dimensions of urban perception, including wealthy, boring, depressing, beautiful, safe, and lively. The participants have three alternatives for reporting their perceptual judgement, namely, left, right, and equal to, indicating their perceptual judgment. Following the comparison, the questions asked, the participants’ choices, and the metadata associated with the street view images (e.g., GPS coordinates). Figure 6 is the conceptual diagram demonstrating the general workflow of the Place Pulse platform.

**Figure:**
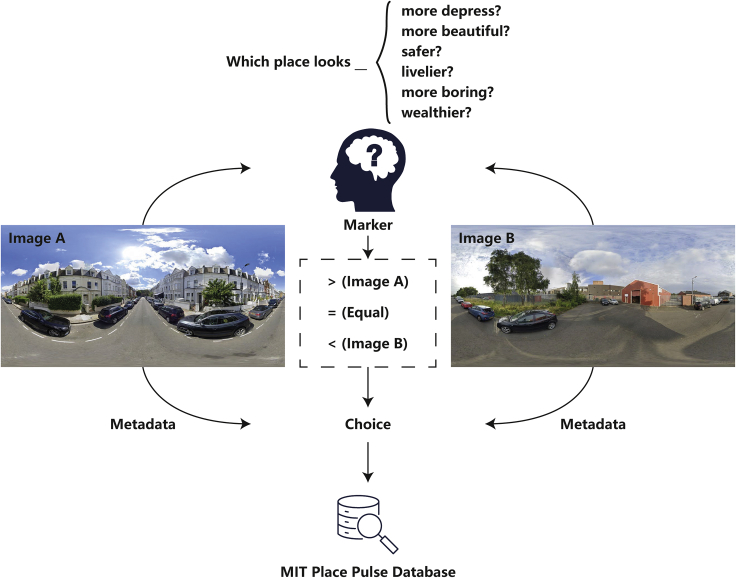
Conceptual diagram of perceptual annotating of street view image for MIT Place Pulse dataset

Place Pulse dataset gathered almost 1.2 million pairwise comparisons from over 80000 online participants on 110988 street view images from 56 cities in 28 countries, which has been assessed without substantial biases for groups with different demographics.^23^^,^^35^^,^^38^ As such, it has been established as ‘the best general dataset covering the worldwide area’ and ‘a perfect training dataset’ for urban perception research.^21^ Numerous previous studies have exemplified the applicability of the Place Pulse dataset to a variety of cities across the globe, including but not limited to China,^23^^,^^49^^,^^50^^,^^51^ the US,^52^^,^^53^ Austria,^35^ and Singapore.^29^ However, the implementation of this dataset in cities in the UK is not well-documented, leading to one of the research objectives of this study is to evaluate such dataset’s utility in the UK context.

#### Proposed analytical framework

Given the aforementioned inadequacies in prior research, this study proposed an analytical framework for extracting neighbourhood-level perception from SVI in a systematic manner. The proposed framework consists of five steps, including 1) Panoramic SVI acquisition and cleaning; 2) Visual element extraction; 3) Perception Elo rating; 4) Urban perceptions prediction; and 5) Neighbourhood-level aggregation, encompassing the detailed process of SVI analysis from initial data downloading to the final output interpretation. Figure 7 is a flowchart illustrating the overall workflow of the proposed framework, which will be detailed in further depth in the following subsections. Two routes (i.e., model training and model deploying) are included in this workflow, presented by the solid and dashed line, respectively.

**Figure:**
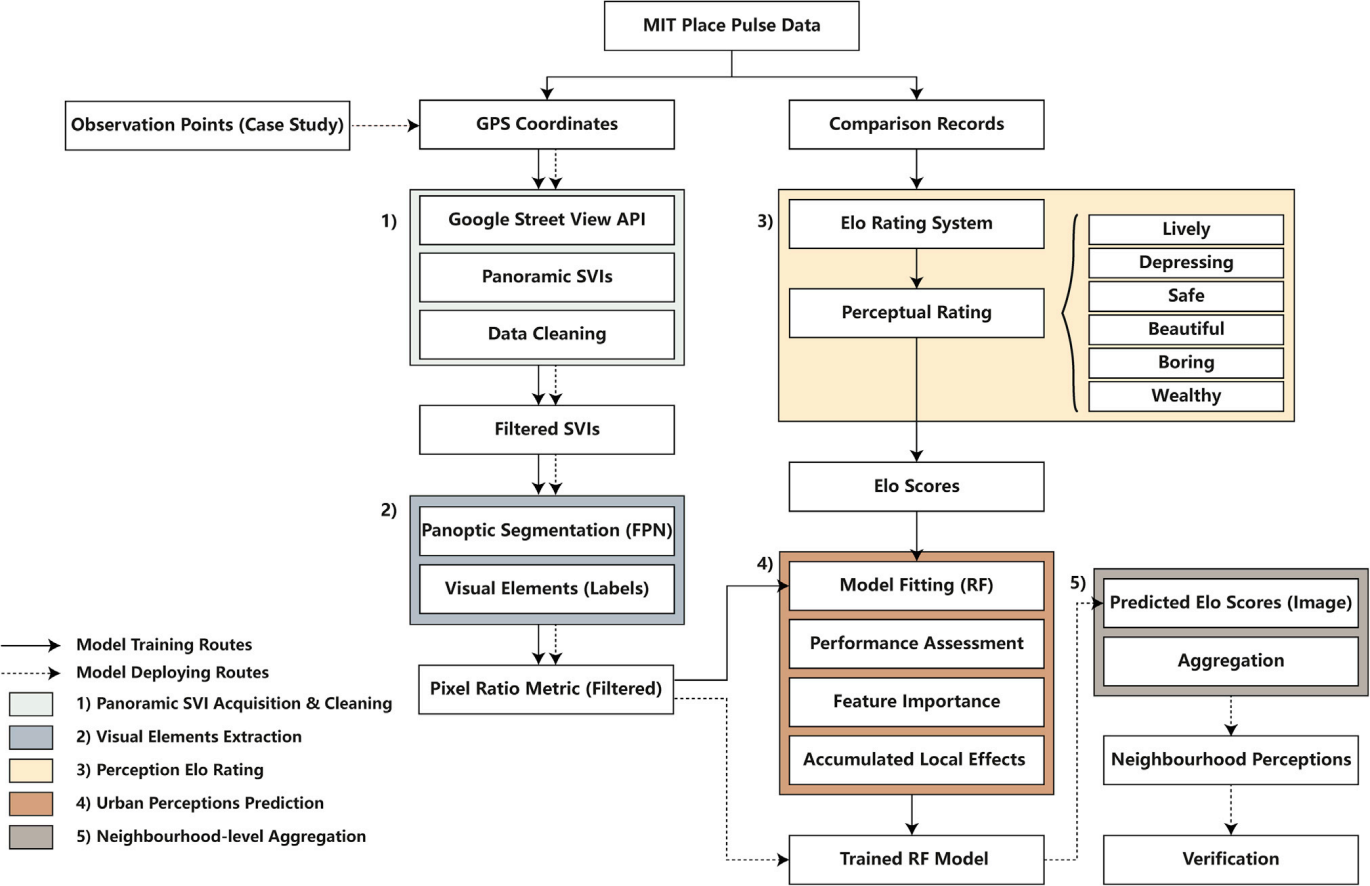
Overall workflow of the proposed analytical framework

The rationale of the proposed framework is to exploit the association between visual elements and human perceptions to develop a machine-learning model for predicting urban perceptions. Specifically, the MIT Place Pulse 2.0 data are divided into two aspects: geo-referenced SVIs and their comparison records, which are imported into Step 2 and 3 for visual element extraction and perception Elo rating, respectively. In Step 2, visual elements in the SVI are extracted and converted into a pixel ratio metric (PRM) by using the panoptic segmentation technique, which is accomplished by implementing a pre-trained panoptic Feature Pyramid Network (FPN). Simultaneously, in Step 3, the Elo rating system is applied to each of the six dimensions of urban perception based on the pairwise comparison history recorded in MIT Place Pulse, computing an Elo score for each SVI that indicates its relative perceptual rating. In Step 4, the PRM and the Elo scores, respectively representing the dependent and independent variables, are used to fit a random forest (RF) regression to train a model that can predict perceptual ratings from visual elements in SVI. Meanwhile, the relationships between the visual elements and six dimensions of urban perception are assessed by feature importance and accumulated local effects (ALE). After the RF model is completely trained and verified, new panoramic SVIs scoured from observation points set in the case study area are fed into Step 2 (i.e., the model deploying routes), followed by the Elo score prediction. In Step 5, these SVIs with predicted perceptual scores are aggregated into neighbourhood-level based on their corresponding geotags, formulating a neighbourhood-level urban perception, followed by an applicability verification stage.

Differing from existing studies that usually adopt the end-to-end structure to predict urban perceptions, the proposed framework utilises human-recognisable visual elements extracted from SVI as an intermediate to predict urban perceptions via a machine-learning model. The reason for this is twofold. First, although recent studies have significantly improved prediction accuracy, interpretability is still one of the most significant gaps in the existing research. Due to the ‘black-box’ nature, most visual features extracted from SVIs are not human-recognisable, and their associations/contributions to human perceptions are concealed in the end-to-end model structure, considerably limiting research usefulness in practice. The proposed framework can considerably improve the feature interpretability since tangible visual elements were extracted from a pre-trained panoptic segmentation model. Moreover, through the built-in feature importance of the RF model and ALE plots, the association between visual elements and urban perceptions can be clarified and visualised, further enhancing the feature interpretability and result interpretability, facilitating more evidence-based decision-making from urban planners or policymakers.

Second, because the seamless panoramic GSVs were stitched from multi-angle SVIs by using image blending techniques,^10^^,^^54^ unlike normal view SVIs, the potential data quality issues in panoramas might provide exceeding difficulties in the training process of the standard end-to-end models, hindering them from learning from the images effectively. However, in the proposed framework, introducing human-recognisable visual elements as an intermediate to simplify the urban perception prediction task can efficiently alleviate the negative impacts of the ‘noisy’ SVIs without significantly sacrificing model complexity and prediction accuracy. Additionally, the pixel ratio metric obtained from the panoptic segmentation model may be used to evaluate the data quality of a given panoramic SVI, assisting analysts in fine-tuning the data for model training, hence improving the prediction accuracy. This is because some abnormal labels (i.e., objects unrelated to street view), or area ratios that may be subject to visual blocking can be easily spotted by the process.

##### Panoramic SVI acquisition and cleaning

Since GPS coordinates had already been stored in the MIT Place Pulse data, the metadata of the corresponding panoramic SVI can be directly acquired by requesting GSV Application Programming Interface (API), subject to data availability. Prior to downloading, parameters were appropriately adjusted to ensure the downloaded images with the same resolution (1024∗512), zoom level, heading, and pitch. Figure 8 displayed some panoramic SVIs randomly sampled from the downloaded data.

**Figure:**
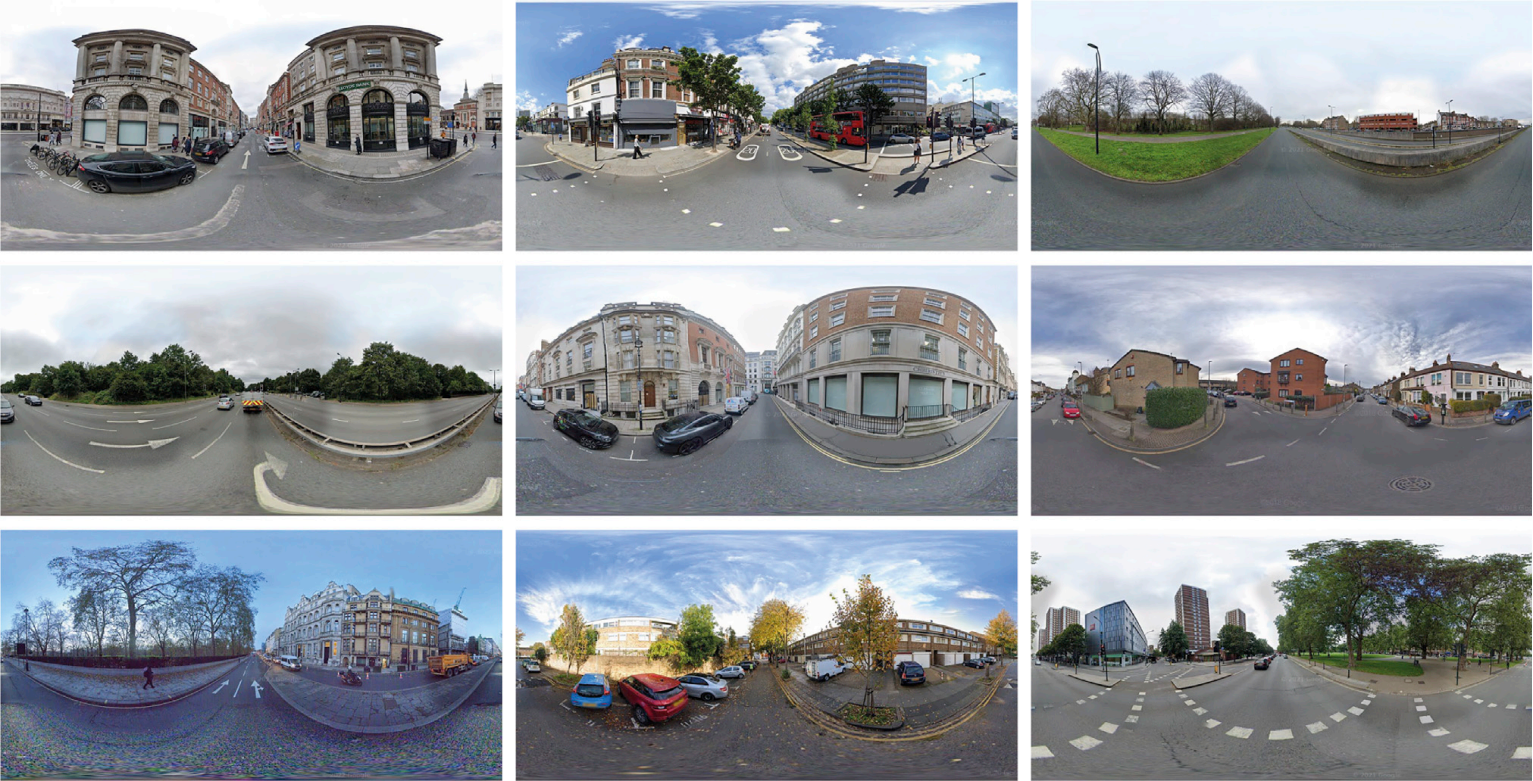
Examples of Panoramic SVIs obtained from GPS coordinates in the MIT Place Pulse 2.0 data

After SVIs were downloaded, data cleaning was undertaken to ensure the data quality, mainly requiring a semi-manual inspection. This inspection aimed to exclude certain typical outliers (i.e., erroneous SVIs) from the dataset used for subsequent steps, despite the filtered data being far from error-free. Several SVIs meeting one of the following filtering criteria were excluded from the dataset: 1) images with abnormal lighting or shading (e.g., overexposed or underexposed); 2) images have significant distortions or noises; 3) views are occluded by objects or blurred by bad weather; 4) images with large-area intentionally blur, glitches, or screen tearing; 5) image resolution or file size being much lower than the average standard; 6) non-street-view image collection (e.g., interior image). Figure 9 exemplifies some typical outliers in the downloaded SVIs.

**Figure:**
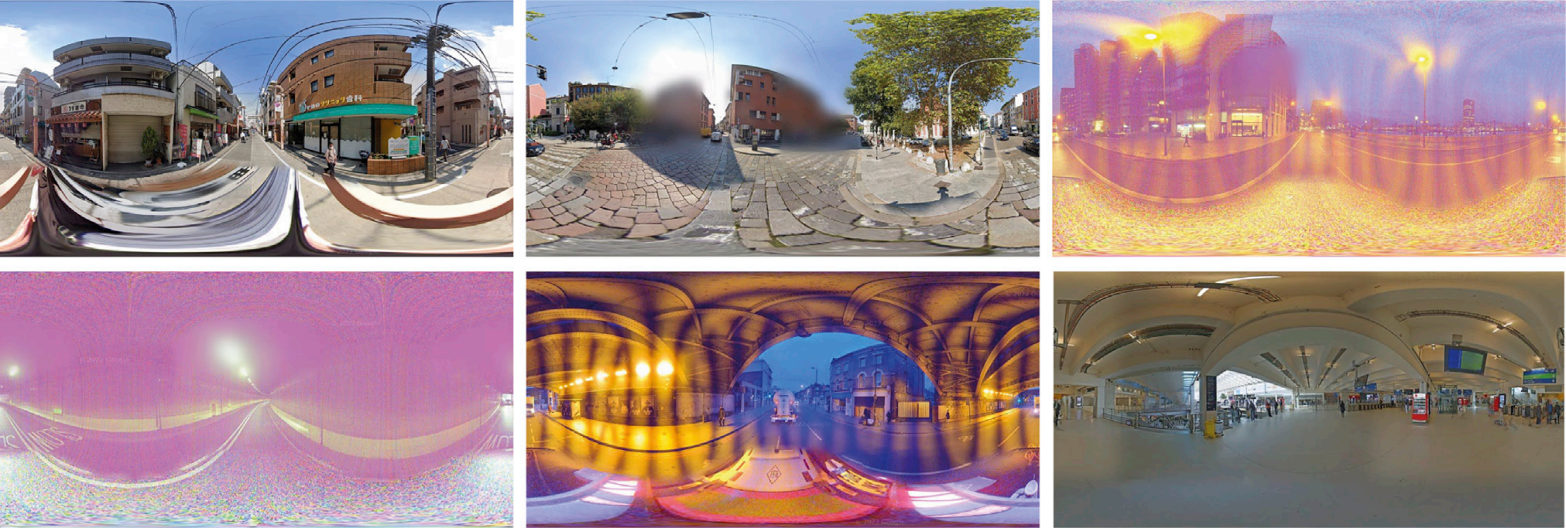
Examples of “noisy” SVIs detected from the downloaded data

By loading 108860 GPS coordinates stored the MIT Place Pulse data into the aforementioned workflow, 99884 panoramic SVIs were finally retained. These SVIs have attached the record of the pairwise comparison, assembling the dataset for the subsequent analysis.

##### Visual elements extraction

To better extract the visual elements from SVI in the real-world version perspective, the panoptic segmentation (PS) technique was utilised in this step. PS represents one of the latest breakthroughs in computer vision, which can be considered as an integration of two traditional image segmentation tasks, namely, semantic segmentation task (i.e., labelling each pixel with a class) and instance segmentation task (i.e., detecting and segmenting each object instance).^55^^,^^56^^,^^57^ Compared to these single-task image segmentation techniques, PS, as a hybrid method, allows the detection of countable objects and uncountable regions simultaneously, providing a complete description of the given image. Therefore, the outputs of PS are more relevant to real-world applications and more in line with human vision and observation, offering a more comprehensive understanding of the environment. Recently, PS has been successfully utilised in autonomous driving,^58^ remote sensing images mapping,^59^ and biomedical images analysis,^60^ with research popularity on rise.

To implement PS to the assembled SVI data, we employed a pre-trained Panoptic Feature Pyramid Network (FPN) model from the Detectron2 platform (https://github.com/facebookresearch/detectron2), a state-of-the-art AI platform for computer vision tasks developed by the Facebook AI research team in 2019. Panoptic FPN, developed by Kirillov et al.,^61^ is one of the latest baseline models for PS tasks with ‘simple, flexible, and effective architecture’ for holistic scene understanding.^61^ Panoptic FPN is a single network utilising ResNet-FPN as the backbone to extract multi-scale features from images,^57^ where ResNet^62^ serves as the encoder and FPN^63^ as the decoder. This pyramid model contains two branches, namely, the instance segmentation branch and the semantic segmentation branch, which generate outputs simultaneously utilising a shared backbone. The instance segmentation branch is based on the output result of Mask R-CNN, generating region-based outputs; and the semantic segmentation is performed on the obtained multi-scale feature maps, generating dense-pixel outputs. More detailed model structure and parameter settings of the panoptic FPN have been documented in,^61^^,^^63^ which will not be further discussed here. Figure 10 demonstrates the structure of this pre-trained Panoptic FPN model and its implementation in panoramic SVIs.

**Figure:**
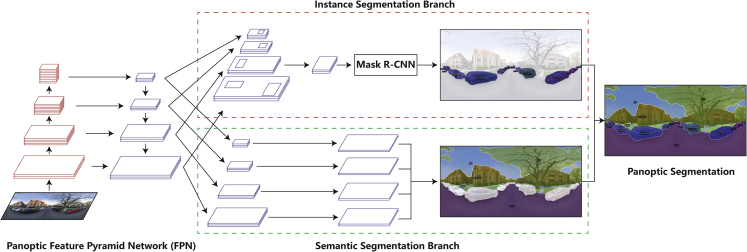
Conceptual diagram of the Panoptic FPN

By applying the Panoptic FPN model to the downloaded SVIs, each pixel in the SVI was labelled (Figure 11). Accordingly, the proportion of area for each visual element in the SVI was obtained by calculating the ratios of the pixels categorised as them over the total number of pixels in the SVI, formulating a pixel ratio metric (PRM) for every SVI. This metric can be further linked to the prediction of urban perceptions in the subsequent steps. In the MIT Place Pulse data, more than 115 labels were identified through the PS. In order to mitigate the potentially negative effects of outliers (e.g., errors from mislabelling and noise data), only frequently occurring labels were retained to construct the PRM. This was achieved by establishing an empirical threshold to filter out random labels that may be attributed to algorithmic error, poor data quality, or contingency. The threshold was set to around 1% of the total SVIs in the prepared dataset, meaning that the retained labels should appear in at least 1000 SVIs. As a result, 19 labels were finally retained to formulate the PRM used in the subsequent analysis.

**Figure:**
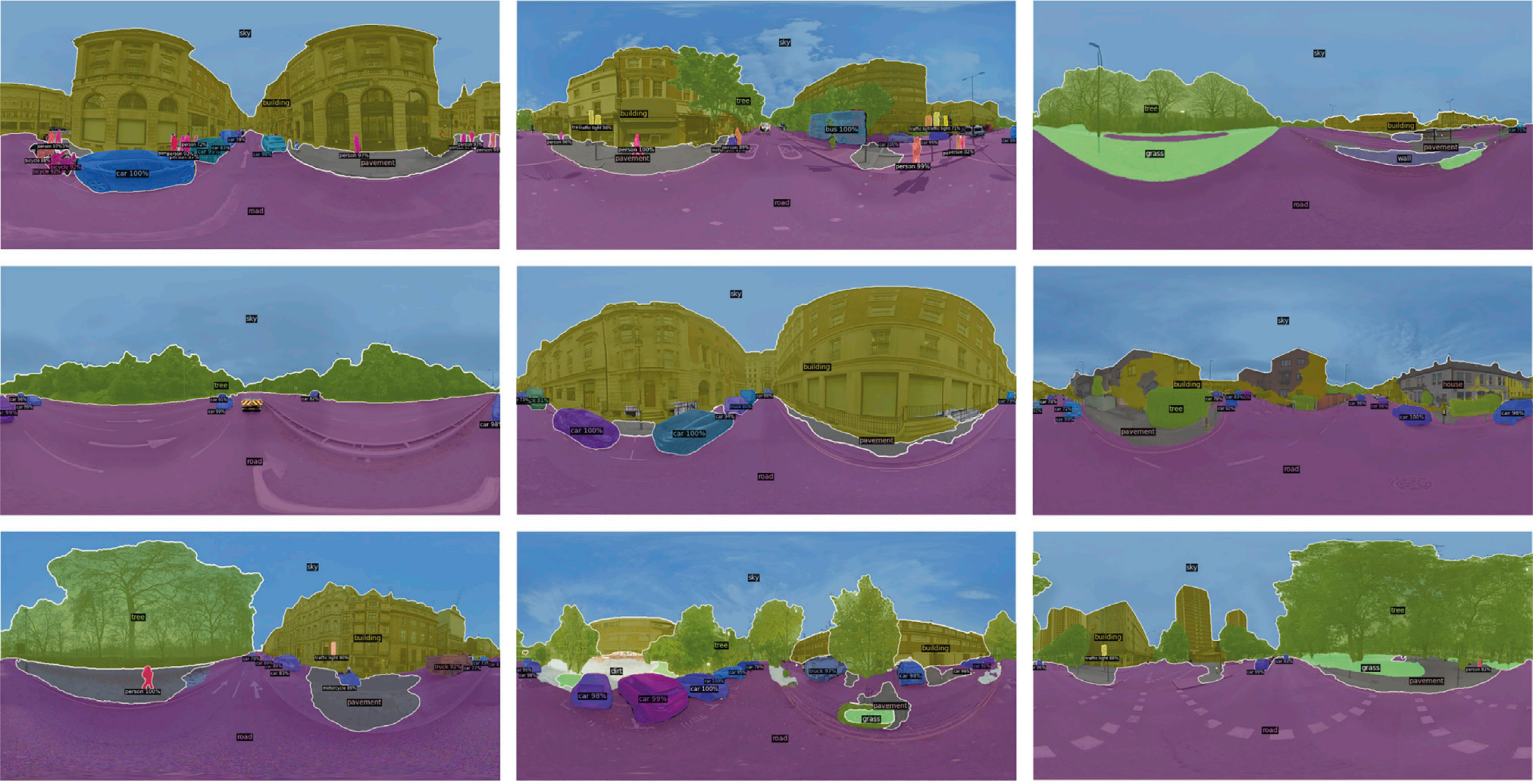
Panoptic segmentation results of the SVI samples

Additionally, the panoptic FPN enables the further examination of SVIs with relatively poor quality or inappropriate content. For example, if a single car in the SVI occupies a vast area of the whole image, such SVI will be spotted and further inspected manually to determine if the image has a problem. SVIs with large-area blocking, distortions, or noises detected through this process will be excluded from the subsequent analysis. Moreover, SVIs in MIT Place Pulse data with several labels that are not usually associated with street views, such as laptops, cups, and cell phones, are also excluded.

##### Perception rating

Although each pairwise comparison recorded in the MIT Place Pulse data may be viewed as a qualitative choice (i.e., victory, loss, or draw), their combination is a relative degree rather than an absolute classification. For instance, if a certain SVI ‘win’ several times because its depicted urban streetscape appears safer than others, it is inappropriate to conclude that this SVI is safe and its rivals are hazardous. Therefore, the original structure of the MIT Place Pulse dataset is inapplicable to the extraction of urban perceptions unless it is quantified. Inspired by the player ranking system widely used in chess, football, and esports, we employed the Elo rating algorithm to generate ‘Elo scores’ from these match histories, thereby quantifying urban perceptions systematically.

The Elo rating system, initially introduced by Arpad Elo in the 1960s,^64^^,^^65^ is a widely used ranking method for calculating the relative skill levels of players in zero-sum games, such as chess and football.^66^ A player’s Elo rating is measured by Elo scores that may change according to the outcome of a match. After every rated match, the victorious player takes a certain number of scores from the losing one within a fixed range, determined by the difference between their respective Elo scores. This system is advantageous for its self-correcting capability as it can continuously update its value depending on the sequence in which comparisons are made.^67^ Accordingly, in the long term, underrated and overrated players are expected to perform better or worse correspondingly than the rating system predicts and hence gain or lose scores until the Elo ratings reflect their actual strength.

Since a complete comparison matrix is not the prerequisite of the Elo rating system and can be acquired at any point for convenient monitoring, this system has been promoted in ecological studies by Neumann et al.^67^ More recently, the Elo rating system has been applied as a scientific analysing tool for transportation analysis,^68^ medical research,^69^ and urban landscape study.^70^ Given the incomplete nature of the pairwise comparisons in the MIT dataset, the Elo rating system is feasible for urban perception rating in this study. Therefore, each panoramic SVI can be considered as a player competing in the arena formed by the pairwise comparisons in MIT Place Pulse data. The Elo rating score can be calculated via the following formula (see Equations 1 and 2).

(Equation 1) $$\begin{array}{c} E_{A}=\frac{1}{1+10^{\frac{\left(R_{B}-R_{A}\right)}{400}}} \end{array}$$

(Equation 2) $$\begin{array}{c} R_{A}^{\prime }=R_{A}+K\left(S_{A}-E_{A}\right) \end{array}$$

where A and B is the notation of a panoramic SVI A and B, respectively; E is the expected winning rate (the initial rate is 0.5); R is the current Elo scores (the initial score is 1500); $R^{\prime }$ is the updated Elo scores after a pairwise comparison; S is the actual matching result, which either be win (1), draw (0.5), or lose (0). K refers to K-Factor indicating a cap on how many Elo scores an image can win or lose from a single comparison, which was set to 32.

As mentioned previously, the MIT Place Pulse dataset contains six types of questions capturing six dimensions of urban perceptions – depressing, beautiful, safe, boring, lively, and wealthy. The Elo rating system was respectively applied to each of these dimensions to generate the Elo scores based on the outcomes of the pairwise comparisons. Given the self-correcting feature, for each perceptual dimension, the rating system was fed the whole matching history to compute the Elo scores. However, to alleviate negative impacts from randomness on the subsequent predictive analysis, the Elo scores of SVIs whose cumulative frequency of comparing exceeded a pre-defined threshold were only retained within the system. This cut-off point was set by the K-means clustering algorithm (k = 2), classifying SVIs into a reserved or unreserved group based on their cumulative comparison frequency. The same approach has been applied to determine infrequent and frequent passengers in the transportation study,^71^ approving its utility in filtering out randomness. Figure 12 shows some SVIs exemplifying the results generated by the Elo rating system.

**Figure:**
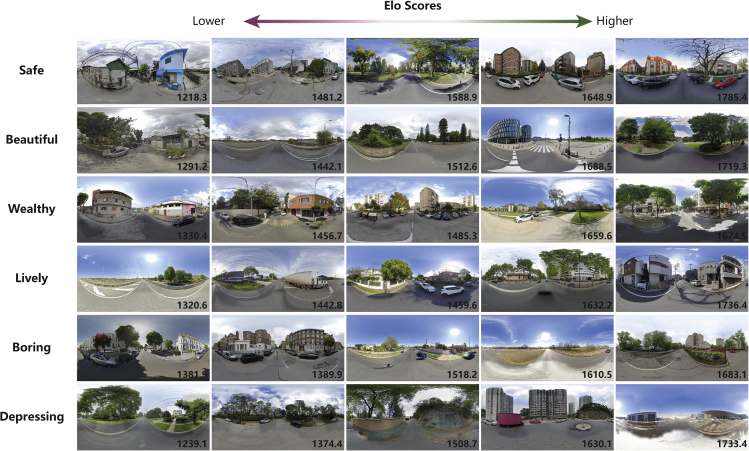
Example results of Elo rating based on pairwise comparisons in MIT Place Pulse dataset

##### Urban perceptions prediction

The Elo scores and PRM were integrated into the Random Forest (RF) algorithm to train the model for urban perception prediction. RF is an ensemble machine learning algorithm for classification and regression tasks that uses averaging to improve prediction accuracy and control overfitting by fitting a number of decision trees to the dataset on various subsamples.^72^ RF was used in this study not only because of its robust performance in model fitting, such as high accuracy and robustness to outliers,^73^^,^^74^ but also due to its advantages in model and feature interpretability,^75^ which uses its built-in feature importance to measure which variables contribute more to the model.

#### Model fitting

To fit the RF model, the PRM and the Elo scores were considered independent and dependent variables, respectively. In each perceptual dimension, 5-fold cross-validation (CV) is applied to evaluate the model performance based on the previous practice.^75^^,^^76^ K-fold CV is a strategy to split data into k number of subsets, using one subset as the validation set while the remaining as the training set at each iteration of k number of times. This procedure generates a more accurate representation compared to single training, validation, and test split.^77^ Before the model training phase, hyperparameter tuning is performed to search for the optimal model architecture. Random search is employed for its high efficiency and low computational requirements. Two key parameters to adjust in this study are the number of trees (n_estimators) and the maximum depth of the tree (max_depth) (The values for hyper parameters that we use include: RF: n_estimators = 200, max_depth = 30;). The hyperparameters can be assigned either a distribution of possible values or a list of discrete values for an adequate set of values. The number of 50 iterations and 5-fold cross-validation are set to obtain a reasonably decent set of values of the hyperparameters for a wider search coverage and further overfitting reduction.

#### Accuracy assessment

To assess the performance of the fitted RF regression models, two metrics, mean absolute error (MAE) of Elo scores and R squared score (R^2^), are utilised to quantify the accuracy between the predictions and the true observations. MAE (Equation 3) identifies the average absolute error or loss between the predicted and actual values, which are always positive and represent better predictions as they become smaller. R^2^ (Equation 4) measures how much of the variance in the output can be explained by the independent variables, ranging from 0 to 1, with higher values representing more explanatory models.

(Equation 3) $$\begin{array}{c} MAE=\frac{1}{n}\sum_{i=1}^{n}\left|y_{i}-{\hat{y}}_{i}\right| \end{array}$$

(Equation 4) $$\begin{array}{c} R^{2}=1-\frac{\Sigma_{\mathrm{i}=1}^{n}{\left(y_{i}-{\hat{y}}_{i}\right)}^{2}}{\sum_{i=1}^{n}{\left(y_{i}-\bar{y_{i}}\right)}^{2}} \end{array}$$

where $y_{i}$ is the ground-truth Elo scores, $\bar{y}$ is equal to $\frac{1}{n}\sum_{i=1}^{n}y_{i}$, and $\hat{y}$ is the predicted Elo scores from the trained RF model.

#### Feature importance interpretation

One of the overarching objectives of this study is to explore the connection between visual elements in SVI and people’s perceptions. As such, RF-based permutation feature importance (PFI), first introduced by,^72^ is employed to discern which visual element contributes more to each dimension of six urban perceptions. PFI measures the increase in its prediction error after the feature has been permuted. Generally, if a given independent variable (i.e., visual element) exhibits a higher PFI value, it has a more statistically significant impact on the dependent variable (i.e., perceptual score). Compared to traditional RF Gini importance (i.e., mean decrease impurity), PFI is more reliable and less biased, particularly in the variable selection process.^78^^,^^79^

#### Accumulated local effects (ALE) plots

To further explore and interpret how the important visual features influence each dimension of urban perceptions, accumulated local effects (ALE) plots are used in this research. ALE plots present details on how variables affect the prediction of a supervised learning model on average,^80^ which is superior to fundamental correlation analysis that only describes the strength and direction of the relationship between two variables. ALE plots are fast, unbiased and can promote the interpretability of ‘black-box’ issues of machine learning models.^79^^,^^80^ In the core of ALE, a feature is divided into several intervals based on its quantiles and the averaged difference in the predictions for a certain interval is calculated to estimate its local effect. By accumulating these local effects of all intervals and centring around zero (i.e., the mean effect is zero), ALE values are obtained and interpreted as the main effects of the feature at certain values compared to the average prediction.

##### Neighbourhood-level aggregation

After the RF model is completely trained and verified, new panoramic SVIs gathered from the observation points established in the case study area can be imported into the analytical framework to generate perceptual scores. Each SVI is assigned six predicted Elo scores indicating six dimensions of urban perceptions. Subsequently, according to their corresponding location, these SVIs were aggregated to neighbourhood-level geography by calculating the average value, for example, Output Area level in the UK or Census Tract level in the US.

The reason for conducting neighbourhood-level aggregation is threefold. First, neighbourhood-level aggregation is more useful than the commonly used street-level aggregation in urban planning practice since it allows researchers to have a more informative and holistic understanding of the urban environment at a large scale without too many detailed complexities. Second, neighbourhood-level aggregation helps smooth out the prediction result without sacrificing too much prediction accuracy or the spatial heterogeneity of the urban perception. This is because even the most cutting-edge prediction model is not error-free in practice, and the inevitable noise input data might further amplify such errors (e.g., images with errors or poor quality). Last but not least, following the rapid development of big data, the concern of geoprivacy has attracted many researchers.^41^^,^^42^ The data availability is less likely to provide a resolution lower than the neighbourhood level, especially for the open data. Therefore, neighbourhood aggregation could improve the urban research’s applicability of outreaching external data, facilitating interdisciplinary cooperation for verification and application.

#### Case study: Model deployment in Inner London areas

To evaluate the utility of the developed model, Inner London boroughs (Figure 13) were selected as the case study area for model deployment. According to the statistical definition provided by Office for National Statistics,^81^ Inner London consists of 14 local authorities, including Hammersmith and Fulham, Kensington and Chelsea, Westminster, City of London, Camden, Haringey, Islington, Hackney, Tower Hamlets, Newham, Wandsworth, Lambeth, Southwark, and Lewisham.

**Figure:**
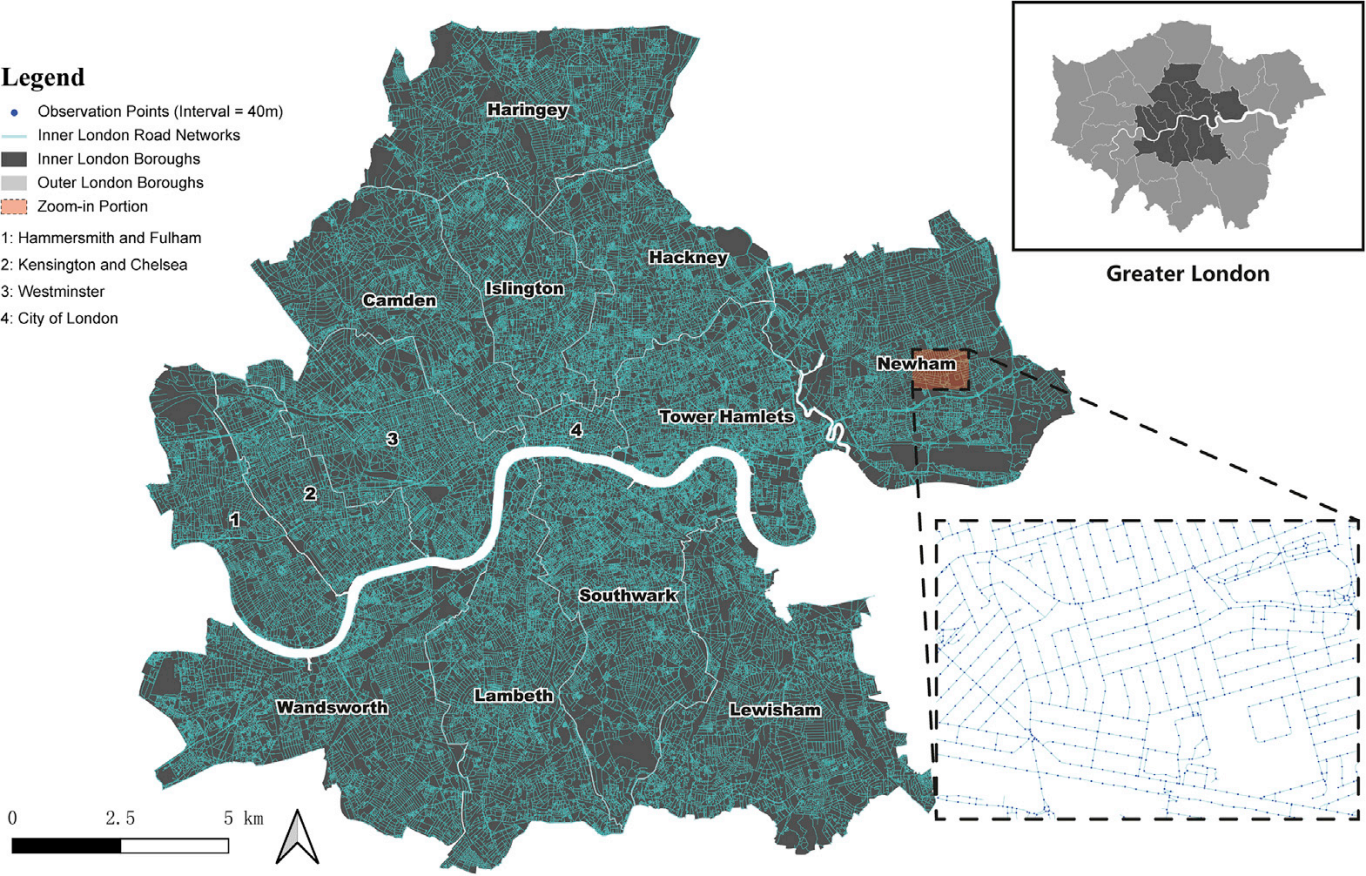
Inner London Boroughs and Observation Points (OPs) along the road network

One of the primary reasons for this selection is London’s distinctive traits – it is the capital and largest city in the UK and the leading global finical centre. As the centre of this metropolitan region, Inner London has the most vibrant human activity, dense population, socioeconomic diversity, and complicated urban fabric. Understanding urban perceptions of this area has several practical significances, assisting urban analysts in obtaining insights into its complexity and heterogeneity and promoting better evidence-based decision-making. Moreover, to the best of our knowledge, this is the first implementation of a large-scale urban perception model in the UK context, bridging the research gap in the existing literature.

##### Obtaining SVIs in Inner London areas

Road network data for Inner London areas were obtained from the Ordnance Survey (OS) (https://digimap.edina.ac.uk/roam/download/os). For each road in the range of the study area, observation points (OPs) for capturing the panoramic SVI were set based on a fixed distance interval (i.e., 40m) along the road centreline, and the road intersections were also used OPs (Figure 13). This distance was partially determined based on the median length of the road segments in the Inner London areas (∼42.5m), with further consideration of the size of the SVI data and computational capacity. The interval segmentation was only applicable to those road segments longer than 40m, while for those shorter, their centroid was used as the OP. Moreover, OPs that are too close to each other were excluded in order to prevent data duplication. Totally, 147529 OPs were established in the study area for downloading panoramic SVIs.

Panoramic SVIs were obtained through querying from GSV API with the GPS coordinates of the OPs (not all OPs offer downloadable GSV images, which may be due to the limited accessibility for GSV vehicles, such as paths in parks or pedestrian-only roads). Following the data cleaning process described in the proposed framework, eventually, 122733 panoramic SVIs were retained in Inner London for urban perception prediction.

##### Applicability verification

One of the key objectives for neighbourhood-level aggregation is to improve the application of urban perceptions by relating them to external urban data. Therefore, an additional correlation analysis is conducted in the same neighbourhood geography for applicability verification.

The real-world OA-level crime rate (Crime data are available at https://data.police.uk/; Only theft crime is used here; The OA-level crime rate is measured by the ratio of crime number and population number at each OA) for Inner London areas is used as an example to examine its relationship with the six dimensions of predicted urban perceptions. To guarantee that the crime rate utilised in the case study is reasonably up-to-date, reliable and steady, the annually averaged crime rate at the OA level is calculated using crime and population data from 2015 to 2019. Figure 14 presents the spatial distribution of the calculated crime rate in the Inner London areas.

**Figure:**
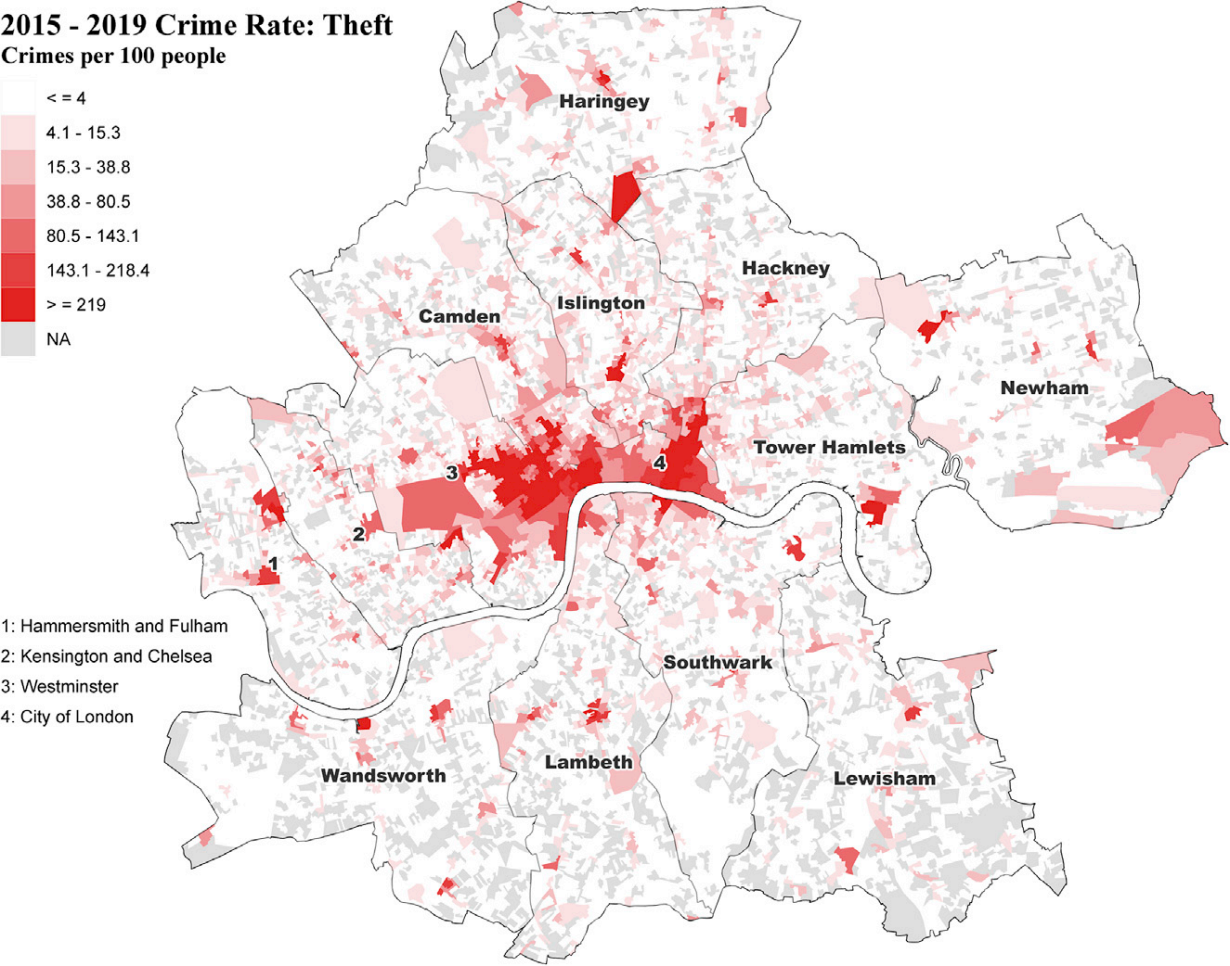
2015-2019 annually averaged OA-level theft crime rate in Inner London areas (crimes per 100 people)

### Quantification and statistical analysis

In this research, we analysed the data using Python, RStudio and QGIS. The panoptic segmentation (PS) was achieved by using the pre-trained FPN model developed by Detectron2 platform which was run under Python environment. After that, the pixel ratio metric (PRM) was calculated using Python. By using the pairwise comparison history stored in the MIT Place Pulse dataset, the perceptual scores for each SVI were calculated by the Elo rating system using RStudio (‘EloRating’). Random forest (including the model training and model accuracy assessment, e.g., MSE) and the built-in feature importance were calculated using Python (‘Scikit-learn’). Accumulated local effects (ALE) plots were generated by using Python (‘ALEPython’). The neighbourhood-level (i.e., OA) perceptual scores were calculated by aggregating the average value of the perceptual scores located within the same OA using spatial join function in QGIS. 2015-2019 theft crime rate for Inner London areas (OA level), and its Spearman correlation coefficient between the urban perceptions (all six dimensions) were calculated by using Python (‘Pandas’).

## Data Availability

• This paper analyses existing, publicly available data. These accession numbers for the datasets are listed in the key resources table. • The codes are available on reasonable request from the lead contact. • Any additional information required to reanalyse the data reported in this paper is available from the lead contact upon request.
